# Association of Household Exposure to Primary *Clostridioides difficile* Infection With Secondary Infection in Family Members

**DOI:** 10.1001/jamanetworkopen.2020.8925

**Published:** 2020-06-26

**Authors:** Aaron C. Miller, Alberto M. Segre, Sriram V. Pemmeraju, Daniel K. Sewell, Philip M. Polgreen

**Affiliations:** 1 Department of Epidemiology, University of Iowa, Iowa City; 2 Department of Computer Science, University of Iowa, Iowa City; 3 Department of Biostatistics, University of Iowa, Iowa City; 4 Department of Internal Medicine, University of Iowa, Iowa City

## Abstract

**Importance:**

*Clostridioides difficile* infection (CDI) is a common hospital-acquired infection. Whether family members are more likely to experience a CDI following CDI in another separate family member remains to be studied.

**Objective:**

To determine the incidence of potential family transmission of CDI.

**Design, Setting, and Participants:**

In this case-control study comparing the incidence of CDI among individuals with prior exposure to a family member with CDI to those without prior family exposure, individuals were binned into monthly enrollment strata based on exposure status (eg, family exposure) and confounding factors (eg, age, prior antibiotic use). Data were derived from population-based, longitudinal commercial insurance claims from the Truven Marketscan Commercial Claims and Encounters and Medicare Supplemental databases from 2001 to 2017. Households with at least 2 family members continuously enrolled for at least 1 month were eligible. CDI incidence was computed within each stratum. A regression model was used to compare incidence of CDI while controlling for possible confounding characteristics.

**Exposures:**

Index CDI cases were identified using inpatient and outpatient diagnosis codes. Exposure risks 60 days prior to infection included CDI diagnosed in another family member, prior hospitalization, and antibiotic use.

**Main Outcomes and Measures:**

The primary outcome was the incidence of CDI in a given monthly enrollment stratum. Separate analyses were considered for CDI diagnosed in outpatient or hospital settings.

**Results:**

A total of 224 818 cases of CDI, representing 194 424 enrollees (55.9% female; mean [SD] age, 52.8 [22.2] years) occurred in families with at least 2 enrollees. Of these, 1074 CDI events (4.8%) occurred following CDI diagnosis in a separate family member. Prior family exposure was significantly associated with increased incidence of CDI, with an incidence rate ratio (IRR) of 12.47 (95% CI, 8.86-16.97); this prior family exposure represented the factor with the second highest IRR behind hospital exposure (IRR, 16.18 [95% CI, 15.31-17.10]). For community-onset CDI cases without prior hospitalization, the IRR for family exposure was 21.74 (95% CI, 15.12-30.01). Age (IRR, 9.90 [95% CI, 8.92-10.98] for ages ≥65 years compared with ages 0-17 years), antibiotic use (IRR, 3.73 [95% CI, 3.41-4.08] for low-risk and 14.26 [95% CI, 13.27-15.31] for high-risk antibiotics compared with no antibiotics), and female sex (IRR, 1.44 [95% CI, 1.36-1.53]) were also positively associated with incidence.

**Conclusions and Relevance:**

This study found that individuals with family exposure may be at significantly greater risk for acquiring CDI, which highlights the importance of the shared environment in the transmission and acquisition of *C difficile*.

## Key Points

**Question:**

Is exposure to a family member with *Clostridioides difficile* infection associated with a greater risk for acquiring *C difficile* infection in exposed individuals?

**Findings:**

In this case-control study of 224 818 cases of *C difficile* infection representing 194 424 insurance plan enrollees, having a family member with *C difficile* infection was significantly associated with increased incidence of *C difficile* infection, even after controlling for other factors.

**Meaning:**

Findings from this study suggest that home environment may be a risk factor in the transmission and acquisition of *C difficile* infection.

## Teaser

This case-control study uses data from 2001 to 2017 to assess the transmission of *Clostridioides difficile* infection in family members in a household with another family member with primary *C difficile* infection.

## Introduction

*Clostridioides difficile* infection (CDI) is a common hospital-acquired infection.^1,2^ Transmission of CDI within health care settings and the role of the environment has been a focus of research.^3^ *C difficile* can be recovered from the environment surrounding patients with CDI^4,5^ and from the hands of health care workers.^6,7^ In addition, *C difficile* spores exist in the environment for long periods and are resistant to many cleaning agents.^8^ Yet some whole genome-sequencing–based investigations suggest that symptomatic cases of CDI within health care facilities may not be the source of many CDI cases in nonoutbreak settings^9^ and that asymptomatic *C difficile* carriers may be responsible for some proportion of CDI transmission in health care settings.^10^

CDIs also occur and may be transmitted outside health care settings.^11,12,13,14,15^ Thus, examining potential CDI exposure outside health care settings may help inform efforts to understand the dynamics of CDI transmission. For example, contamination of environmental surfaces with *C difficile* spores has been shown to persist in household settings of patients with documented CDI.^16^ Furthermore, family members and pets have been found to be colonized with *C difficile*.^16,17,18^ Also, some small investigations have identified potential cases of secondary transmission within families.^5,19,20^

Our objective was to determine the incidence of potential family CDI transmission using a large population-based data set. Specifically, we sought to determine whether exposure to CDI in a family member was associated with developing CDI. We used a database of longitudinal insurance claims that allowed linkage of records among family members enrolled in the same plan. The primary outcome was the incidence of CDI in a given monthly enrollment stratum.

## Methods

### Data Source

For this case-control study, we used the Truven Marketscan Commercial Claims and Encounters and Medicare Supplemental databases from 2001 to 2017. These databases represent one of the largest sources of longitudinal insurance claims in the US, containing more than 195 million distinct enrollees or more than 6 billion total enrollment months. The database covers outpatient, emergency, and inpatient visits along with outpatient medications, demographic data, and other enrollment details. Enrollment plan identifiers allow linkage of claims from multiple family members (eg, spouses, children, or dependents) who are enrolled in the same insurance plan. This study is deemed to not be human participant research by the University of Iowa institutional review board, and thus a waiver of informed consent was granted. This study follows the Strengthening the Reporting of Observational Studies in Epidemiology (STROBE) reporting guideline for cohort studies.

### Study Population

Analysis was limited to households with 2 or more family members enrolled in the same insurance plan for an entire month. CDI cases were identified in inpatient or outpatient settings using *International Classification of Diseases, Ninth Revision, Clinical Modification* code 008.45 and *International Statistical Classification of Diseases, Tenth Revision, Clinical Modification* codes A04.7, A04.71, and A04.72. Recurring CDI diagnoses separated by less than 60 days were treated as a single CDI episode,^21,22,23^ with the initial CDI diagnosis labeled as the *index CDI diagnosis*. The 60-day interval was chosen to maximize capture of exposure risk and to avoid attributing recurrent CDI cases to a single case. Most community-onset health care-associated cases occur within 60 days of discharge,^24,25^ and 60 days is commonly used as a window of risk for recurrent cases.^21,22,23^

With a focus on identifying possible family transmission, a distinction was not made between health care– and community–associated cases using traditional surveillance definitions. Rather, for all CDI cases, we account for prior health care and family exposures as separate exposure variables within the statistical models described in the next section. The source of infection/colonization for patients with both family and health care exposure cannot be determined. Thus, we attempt to isolate family transmission by conducting separate analyses for all CDI cases, community-onset cases, and community-onset cases in which enrollees had no prior hospitalization within 60 days.

### Statistical Analysis

For each enrollment month, we assigned individuals to 1 of 4 outcome groups based on CDI status and family exposure to CDI. Enrollees were defined to have *family exposure* to CDI if another family member had any CDI diagnosis during the prior 60 days (regardless of where the prior family member’s index diagnosis occurred). For patients without CDI, we identified family exposure within 60 days prior to the start of the month. For patients with CDI, we considered exposure in the 60-day period prior to the index CDI diagnosis. Thus, in each enrollment month, enrollees were placed into 1 of 4 categories: (1) CDI and prior family exposure, (2) no CDI and prior family exposure, (3) CDI and no family exposure, and (4) no CDI and no family exposure.

Next, we estimated CDI incidence in nonexposed and exposed groups by comparing monthly counts between groups (1) and (2) or between groups (3) and (4), respectively (Figure). Individuals were excluded from analysis (considered not to be at risk) for the 60 days following their index CDI visit and returned to eligibility in the first full month that occurred 60 days following a prior CDI diagnosis.

**Figure.**
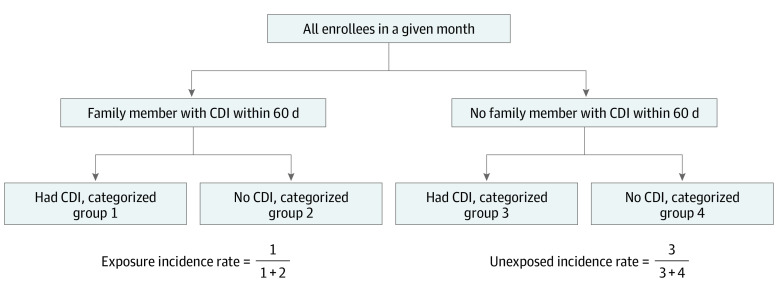
Study Population by Month of Enrollment CDI indicates* Clostridioides difficile* infection.

We used 2 approaches to compare the incidence of CDI in individuals with and without family exposure to CDI. First, we performed a bivariate comparison across multiple confounding factors. Specifically, we compared the incidence rate ratio (IRR) between exposed and nonexposed individuals by age group, sex, prior antibiotic exposure, prior hospitalization, and year. For prior antibiotic exposure, we considered prescriptions filled within 60 days prior to the CDI diagnosis or enrollment month. We created separate categories for high risk of CDI antibiotics (ie, clindamycin, fluoroquinolones, or cephalosporins) vs low risk of CDI antibiotics (ie, penicillins, macrolides, sulfonamides, or trimethoprim), as the antibiotics with a high risk of CDI have been shown to pose the greatest risk for community-associated CDI.^13,26^ For prior hospitalization, we identified prior hospitalizations that occurred within 60 days before the CDI diagnosis (or the start of the enrollment month for enrollees without CDI).

For our second approach, we used a regression model to estimate the monthly incidence of CDI associated with prior exposures and patient characteristics. We controlled for confounding characteristics by binning enrollees into monthly enrollment-strata by month, year, age, sex, prior antibiotic use, prior hospitalization, and prior family exposure to CDI. Only patients enrolled for the entire month in a given monthly enrollment-stratum were included. The CDI incidence in each monthly enrollment-stratum was then regressed on the risk factors that defined the strata. Specifically, for each stratum, the dependent variable was the CDI incidence count (number of CDI cases) in that stratum. The independent variables were binary indicator variables for family exposure (exposed or nonexposed) and each of the other risk factors used to define the strata (ie, year, month, sex, age group, prior antibiotic use, and prior hospitalization). We estimated CDI incidence using a log-linear generalized linear regression model. We used a quasi-Poisson distribution to account for overdispersion with this formula:

log(*Mean CDI Count_j_*) = α + *X_j_β* + log(*enrollment_j_*),

where *Mean CDI Count_j_* is the expected count of CDI cases in stratum *j*, *X_j_* is a vector of indicators for each factor used to define stratum *j* (eg, age group, prior hospitalization, or prior family exposure), *enrollment_j_* is the number of person-days of enrollment in stratum *j* (acting as an offset to account for differences in enrollment across strata), and α and β are parameters to be estimated.

We separated age groups into 5 bins: ages 0 to 17 years, 18 to 26 years, 27 to 44 years, 45 to 64 years, and 65 years or older. Because dependent enrollees between the ages of 18-26 often live outside of the household of the primary enrollee (eg, children in college), we created separate age-dependency strata for dependents aged 18 to 26 years vs primary enrollees or spouses aged 18 to 26 years. The Truven Marketscan Commercial Claims and Encounters data set contains indicators for primary employee, spouse, child, or dependent. We then interacted the dependent age 18-to-26-years strata with the prior-family-exposure strata to estimate separate family exposure results for these groups. We hypothesized that the association of family exposure with CDI may be lower for the dependent or age group potentially living outside the household (ie, dependents age 18-26 years).

### Validation and Sensitivity Analysis

We do not have access to laboratory data to confirm CDI diagnoses, so we performed a sensitivity analysis to increase the specificity of outpatient CDI case identification. For outpatient CDI, we identified subsequent outpatient antibiotic treatment (ie, oral metronidazole or vancomycin) or subsequent CDI hospitalization within 30 days of the initial diagnosis. We then excluded outpatient-only CDI cases without evidence of appropriate antibiotic treatment or subsequent CDI hospitalization.

## Results

Table 1 summarizes the baseline enrollment characteristics of the study population. In total, there were 224 818 cases of CDI, representing 194 424 (1.4%) enrollees (55.9% female; mean [SD] age, 52.8 [22.2] years) that occurred in multiple-enrollee families. Approximately 90 212 (40.1%) cases were hospital onset, initially diagnosed during an inpatient hospitalization, with the remainder being community onset, which may be attributable to either community or hospital exposure to *C difficile*. Of these CDI cases, 1074 events from 1050 individuals (4.8%) followed a prior CDI event in a separate family member, representing possible transmission.

**Table 1.** Baseline Characteristics of Enrollment Families With Multiple Members

Characteristic	No. (%)		
	All enrollees	Episodes of index CDI diagnoses (events occurring ≥60 d prior to another episode)	Possible transmission (following family exposure)
No. of cases	NA	224 818	1074
Hospital onset	NA	90 212	210
Community onset	NA	134 606	864
No. of enrollees	142 125 247	194 424	1050
Age group at enrollment or CDI diagnosis, y			
0-17	47 733 847 (33.6)	19 719 (8.8)	170 (15.8)
18-26	18 209 797 (12.8)	13 963 (6.2)	52 (4.8)
27-44	37 381 120 (26.3)	33 665 (15.0)	143 (13.3)
45-64	34 393 271 (24.2)	89 293 (39.7)	285 (26.5)
≥65	4 407 212 (3.10)	68 178 (30.3)	424 (39.5)
Female sex	71 639 772 (50.4)	125 685 (55.9)	540 (50.3)
Family size			
2	36 598 138 (25.8)	134 644 (59.9)	609 (56.7)
3	29 857 746 (21.0)	36 236 (16.1)	144 (13.4)
4	40 705 784 (28.6)	34 559 (15.4)	210 (19.6)
5	21 536 725 (15.2)	13 517 (6.0)	74 (6.89)
>5	13 426 854 (9.4)	5862 (2.6)	37 (3.45)

Abbreviations: CDI, *Clostridioides difficile* infection; NA, not applicable.

In general, cases of CDI tended to occur in older enrollees (ages 45-64 years and ≥65 years by bin), more frequently in female enrollees, and in smaller-sized households (with only 2 family members). In contrast, CDI cases representing possible transmission events tended to occur in younger individuals (ages 0-17 years by bin) and in households of larger size (with ≥4 members).

Table 2 presents a bivariate comparison of the CDI incidence between individuals with and without family exposure, along with the IRR between these 2 groups. Across all strata, incidence of CDI was greater among individuals with prior family exposure compared with individuals without family exposure. For example, the unadjusted IRR was 8.74 (family exposure CDI incidence per 100 000 enrollment months: 1014.29 vs unexposed CDI incidence per 100 000 enrollment months: 116.07) for prior hospitalization and 56.33 (family exposure CDI incidence per 100 000 enrollment months: 74.36 vs unexposed CDI incidence per 100 000 enrollment months: 1.32) for ages 0 to 17 years and varied across time, with 15.75 in 2016 and 62.56 in 2003. At a bivariate level, CDI incidence was also associated with known risk factors for CDI in both the exposed and unexposed groups: CDI incidence was associated with age (from an incidence of 35.51 per 100 000 enrollment months in ages 18-26 years to an incidence of 266.22 in ages >65 years), female sex (incidence of 107.91 for male individuals and 123.34 for female individuals), prior exposure to antibiotics (incidence of 155.76 for low-risk antibiotics and 666.92 for high-risk antibiotics vs 93.32 for no antibiotics), and hospitalization (incidence of 1014.29 for prior hospitalization vs 100.27 for no prior hospitalization). CDI incidence was also generally increasing across time (from an incidence of 62.77 per 100 000 enrollment months in 2001 to an incidence of 136.77 in 2013).

**Table 2.** Bivariate Comparison of CDI Incidence^a^

Variable	No.						Unadjusted incidence rate ratio
	Family exposure to prior CDI			No family exposure			
	CDI cases	Total enrollee months	CDI incidence (100 000 enrollee months)	CDI cases	Total enrollee months	CDI incidence (100 000 enrollee months)	
Overall	1074	932 669	115.15	223 744	5 118 687 974	4.37	26.35
Age group, y							
0-17	170	228 611	74.36	19 549	1 480 123 664	1.32	56.33
18-26	52	147 277	35.31	13 911	641 617 011	2.17	16.27
27-44	143	131 144	109.04	33 522	1 150 188 683	2.91	37.47
45-64	285	266 371	106.99	89 008	1 542 639 189	5.77	18.54
≥65	424	159 265	266.22	67 754	304 119 427	22.28	11.95
Dependent with ages 18-26 y							
Primary or spouse	1032	791 384	130.4	211 450	4 548 176 692	4.65	28.04
Dependent	42	141 285	29.73	12 294	570 511 282	2.15	13.83
Sex							
Male	534	494 843	107.91	98 599	2 547 683 629	3.87	27.88
Female	540	437 826	123.34	125 145	2 571 004 345	4.87	25.33
Prior outpatient antibiotic use (30-d)							
None	786	842 246	93.32	145 530	4 769 577 359	3.05	30.6
Low-risk antibiotic^b^	96	61 634	155.76	26 819	250 311 054	10.71	14.54
High-risk antibiotic^c^	192	28 789	666.92	51 395	98 799 561	52.02	12.82
Prior (60-d) hospitalization							
No	920	917 487	100.27	164 650	5 067 776 205	3.25	30.85
Yes	154	15 183	1014.29	59 094	50 911 768	116.07	8.74
Year							
2001	2	3186	62.77	1114	60 081 471	1.85	33.93
2002	8	7061	113.3	2168	103 646 351	2.09	54.21
2003	12	9887	121.37	2956	152 275 419	1.94	62.56
2004	20	15 388	129.97	4705	201 717 252	2.33	55.78
2005	31	20 678	149.92	6077	219 601 578	2.77	54.12
2006	38	26 443	143.71	7323	273 882 465	2.67	53.82
2007	27	32 786	82.35	8882	298 099 480	2.98	27.63
2008	47	49 562	94.83	14 186	422 606 758	3.36	28.22
2009	64	66 840	95.75	17 339	460 282 748	3.77	25.4
2010	76	67 809	112.08	17 397	434 093 933	4.01	27.95
2011	115	91 960	125.05	22 664	476 043 269	4.76	26.27
2012	141	109 690	128.54	25 143	485 236 142	5.18	24.81
2013	132	96 509	136.77	22 339	397 177 546	5.62	24.34
2014	111	109 225	101.63	24 418	411 606 877	5.93	17.14
2015	90	77 180	116.61	16 681	251 991 346	6.62	17.61
2016	89	81 794	108.81	16 924	244 800 561	6.91	15.75
2017	71	66 670	106.49	13 428	225 544 779	5.95	17.9

Abbreviation: CDI, *Clostridioides difficile* infection.

^a^ Incidence rates and incidence rate ratios are computed by comparing monthly counts between the CDI and prior family exposure group and the no CDI and prior family exposure group and between the CDI and no family exposure group and the no CDI and no family exposure group.

^b^ Low-risk antiobiotics include penicillin, macrolides, sulfonamides, and trimethoprim.

^c^ High-risk antibiotics include clindamycin, fluoroquinolones, and cephalosporins.

Results of the stratified regression analysis are presented in Table 3. Our regression analysis resulted in a total of 24 793 different demographic/enrollment strata. Across all cases of CDI, prior family exposure was significantly associated with increased incidence of CDI, with an IRR for family exposure of 12.47 (95% CI, 8.86-16.97). This rate represented the covariate with the second highest IRR behind prior hospital exposure (16.18 [95% CI, 15.31-17.10]). Other regression results were also consistent with established risk factors for CDI. Incidence was associated with age and female sex, with an IRR of 9.90 (95% CI, 8.93-10.98) for individuals aged greater than or equal to 65 years compared with individuals aged 0 to 17 years and an IRR of 1.44 (95% CI, 1.36-1.53) for individuals of female sex compared with male sex. Antibiotic exposure was also associated with greater incidence of CDI, and high-CDI-risk antibiotics were associated with more than 2 times the CDI IRR (7.78 [95% CI, 7.33-8.25]) as low-risk antibiotics (3.15 [95% CI, 2.93-3.38]).

**Table 3.** Regression Analysis Incidence Rate Ratios From a Quasi-Poisson Regression Model^a^

Coefficient	Estimate (95% CI)		
	Any CDI (No. of CDI cases = 224 818)	Community-onset CDI (No. of CDI cases = 134 606)	Community-onset CDI and no prior hospitalization (No. of CDI cases = 103 995)
Intercept	0 (0-0)	0 (0-0)	0 (0-0)
Family exposure	12.47 (8.86-16.97)	16.00 (11.72-21.22)	21.74 (15.12-30.01)
Family exposure: dependent (ages 18-26 y)	0.87 (0.10-3.27)	0.97 (0.17-3.06)	0.72 (0.10-2.50)
Prior hospitalization	16.18 (15.31-17.10)	12.73 (11.98-13.52)	NA
Prior antibiotic use			
None	1 [Reference]	1 [Reference]	1 [Reference]
Low-risk antibiotic	3.15 (2.93-3.38)	3.19 (2.96-3.43)	3.73 (3.41-4.08)
High-risk antibiotic	7.78 (7.33-8.25)	8.38 (7.87-8.91)	14.26 (13.27-15.31)
Age group, y			
0-17	1 [Reference]	1 [Reference]	1 [Reference]
18-26	1.54 (1.37-1.73)	1.55 (1.37-1.74)	1.59 (1.39-1.81)
27-44	1.96 (1.79-2.16)	2.02 (1.84-2.23)	2.11 (1.89-2.35)
45-64	3.75 (3.46-4.08)	3.34 (3.07-3.64)	3.22 (2.93-3.55)
≥65	10.34 (9.50-11.28)	11.13 (10.19-12.17)	9.90 (8.92-10.98)
Female sex	1.27 (1.21-1.32)	1.38 (1.31-1.44)	1.44 (1.36-1.53)
Year			
2001	1 [Reference]	1 [Reference]	1 [Reference]
2002	1.29 (0.88-1.90)	1.09 (0.71-1.70)	1.05 (0.60-1.89)
2003	1.32 (0.92-1.92)	1.07 (0.71-1.65)	1.02 (0.60-1.79)
2004	1.59 (1.14-2.28)	1.30 (0.89-1.95)	1.22 (0.75-2.08)
2005	1.83 (1.32-2.60)	1.57 (1.09-2.33)	1.51 (0.94-2.53)
2006	1.96 (1.43-2.78)	1.89 (1.32-2.78)	1.80 (1.14-2.99)
2007	2.21 (1.61-3.12)	1.94 (1.37-2.86)	1.92 (1.23-3.18)
2008	2.58 (1.90-3.62)	2.29 (1.62-3.34)	2.23 (1.44-3.67)
2009	2.96 (2.18-4.14)	3.10 (2.21-4.51)	3.03 (1.97-4.95)
2010	3.17 (2.33-4.44)	3.50 (2.50-5.10)	3.45 (2.25-5.64)
2011	3.75 (2.77-5.24)	4.20 (3.01-6.10)	4.21 (2.75-6.87)
2012	4.17 (3.08-5.82)	4.81 (3.44-6.97)	4.89 (3.20-7.97)
2013	4.38 (3.23-6.12)	5.13 (3.67-7.44)	5.30 (3.47-8.64)
2014	4.90 (3.62-6.84)	5.90 (4.23-8.55)	6.13 (4.02-1.00)
2015	5.04 (3.71-7.06)	6.37 (4.55-9.25)	6.72 (4.39-10.97)
2016	5.34 (3.93-7.48)	7.03 (5.02-10.21)	7.59 (4.96-12.38)
2017	5.01 (3.67-7.03)	6.64 (4.73-9.66)	7.12 (4.64-11.63)
Month			
January	1 [Reference]	1 [Reference]	1 [Reference]
February	0.75 (0.68-0.84)	0.74 (0.66-0.83)	0.69 (0.60-0.80)
March	0.64 (0.58-0.72)	0.64 (0.58-0.72)	0.59 (0.52-0.68)
April	0.65 (0.58-0.72)	0.65 (0.58-0.73)	0.61 (0.53-0.70)
May	0.63 (0.57-0.70)	0.63 (0.57-0.71)	0.60 (0.52-0.69)
June	0.66 (0.59-0.73)	0.67 (0.60-0.75)	0.64 (0.56-0.73)
July	0.65 (0.58-0.72)	0.65 (0.58-0.72)	0.62 (0.54-0.71)
August	0.67 (0.60-0.75)	0.68 (0.60-0.76)	0.65 (0.57-0.75)
September	0.67 (0.60-0.75)	0.68 (0.61-0.76)	0.66 (0.58-0.76)
October	0.68 (0.61-0.76)	0.70 (0.62-0.78)	0.68 (0.59-0.77)
November	0.65 (0.58-0.72)	0.65 (0.58-0.73)	0.63 (0.55-0.72)
December	0.64 (0.57-0.71)	0.64 (0.57-0.71)	0.60 (0.52-0.68)

Abbreviations: CDI, *Clostridioides difficile* infection; NA, not applicable.

^a^ The regression model includes an offset for number of enrollment months. Because the family exposure group was followed for 60 days to identify secondary CDI, the length of their enrollment period is 60 days. For the unexposed group, the length of enrollment was the length of a given month.

Table 3 also summarizes results of the regression analysis when cases of CDI are broken into subgroups less likely to be attributable to hospital exposure (ie, community-onset cases and community-onset cases without hospitalization in the previous 60 days). Family exposure was associated with CDI even in subgroups in which CDI may be less attributable to hospital exposure. The IRR associated with family exposure was 16.00 (95% CI, 11.72-21.22) for community-onset CDI and 21.74 (95% CI, 15.12-30.01) for community-onset CDI without prior hospitalization. In the case of community-onset CDI, family exposure represented the greatest factor in explaining incidence rates and was roughly equivalent to the association with prior hospitalization (IRR, 12.73 [95% CI, 11.98-13.52]). All of the additional risk factor estimates remained generally consistent.

eTable 1 in the Supplement summarizes the results of the sensitivity analysis for community-onset CDI cases validated by antibiotic treatment or subsequent hospitalization. A total of 51 878 of the total 134 606 CDI cases could be validated by subsequent antibiotic treatment or inpatient hospitalization. When these more specific criteria were used to select outpatient cases of CDI, our results remained generally consistent across the different risk factors. The estimated IRR associated with prior family exposure decreased slightly (for example, the odds ratio for community-onset CDI associated with family exposure decreased from 16.00 [95% CI, CI, 11.72-21.22] to 10.69 [95% CI, 6.46-16.46]), but the pattern across subgroups remained consistent: prior family exposure was 8.35 (95% CI, 5.10-12.77) times greater incidence for all CDI cases, 10.69 (95% CI, 6.46-16.46) for community-onset CDI, and 13.26 (95% CI, 6.80-22.93) for community-onset CDI without prior hospitalization.

In addition to the results observed in community-onset CDI, family exposure was still associated with significantly greater incidence of hospital-onset CDI. eTable 2 in the Supplement presents regression results for CDI cases in which symptom onset occurred in a hospital setting. Results are also broken down into hospital-onset CDI cases without prior hospitalization (ie, no hospitalization in the prior 60 days other than the hospitalization when CDI was diagnosed). The IRR for family exposure was 6.73 (95% CI, 3.30-12.00) for hospital-onset cases and 8.87 (95% CI, 3.56-17.89) for hospital-onset cases without previous hospitalization.

The estimated association of prior family exposure on CDI was decreased in dependents aged 18 to 26 years for the primary findings (odds ratios for dependents aged 18-26 years were 0.87 [95% CI, 0.10-3.27] for all CDI cases, 0.97 [95% CI, 0.17-3.06] for community-onset CDI cases, and 0.72 [95% CI, 0.10-2.50] for community-onset CDI cases with no prior hospitalization) (Table 3) and hospital-onset cases (IRR, 0.23 [95% CI, 0.00-6.57] for hospital-onset CDI cases with prior hospitalization and 0.25 [95% CI, 0.00-9.56] for hospital-onset CDI cases without prior hospitalization) (eTable 2 in the Supplement). These results did not reach a level of statistical significance, and the estimates are opposite but nonsignificant for the validated community-onset cases (IRR, 1.41 [95% CI, 0.14-5.83] for community-onset cases with prior hospitalization and IRR, 1.13 [95% CI, 0.06-6.05] for community-onset cases without prior hospitalization) (eTable 1 in the Supplement). These nonsignificant findings may be attributable to the very low number of CDI cases occurring in dependents aged 18 to 26 years.

## Discussion

We found that the incidence of CDI was associated with the diagnosis of CDI in a family member. Specifically, the adjusted incidence among individuals with family exposure to CDI was up to 21 times greater than the incidence among nonexposed individuals. For individuals with family exposure, the risk for being diagnosed with CDI remained consistent after controlling for CDI risk factors and different model specifications. Together, these results suggest that individuals with family exposure may be at greater risk for acquiring CDI than those without exposure and highlight the importance of the shared environment in the transmission and acquisition of *C difficile*. While our results show a relatively high degree of association between family exposure and CDI, the level of risk attributable to household CDI transmission remains low; in our data set, approximately 0.48% of all CDI cases in the population may be attributable to family exposure.

Prior studies have demonstrated the possibility of family transmission in household settings.^15,27^However, the size of our population allowed us to control for a range of potential CDI risk factors. For example, we reported that outpatient antibiotics are a risk factor and that higher-risk antibiotics conferred more risk. We also found that the risk for CDI acquisition is associated with age, consistent with previous CDI-related investigations.^28,29,30^ Furthermore, we observed an overall increase in the incidence of CDI during our study period, as reported elsewhere.^15,31,32^ In addition, the incidence of CDI cases associated with exposure to family members with CDI peaked during the winter, similar to other reports.^33^

The results of our study help clarify *C difficile* transmission. The role of the health care environment has been questioned based on the results of whole genome-sequencing studies.^9^ However, our results suggest that the environment plays an important role in CDI transmission when considering both health care and community settings. Community-onset CDI cases do occur.^15,27^ Our data allow us to determine if and when individuals were exposed to health care settings, and our results clearly suggest that family exposure is associated with the risk for secondary cases of CDI. The large increase in relative incidence associated with family exposure compared with the other risk factors evaluated (eg, antibiotic exposures) highlights the relative importance of the environment in family transmission.

Our results may have practical implications for family members of CDI patients. For example, family exposure followed by diarrhea should suggest CDI testing. Also, from a prevention standpoint, findings emphasize the importance of cleaning shared bathrooms with effective agents. Yet, it is also important to emphasize that the absolute risk of acquiring CDI is low just as it is in the hospital setting.

### Limitations

This study has limitations. One limitation of our study is that the exact size and structure of each family cannot be determined. Family households may have members enrolled in different insurance policies, or enrollees in the same policy may live apart. However, this limitation likely biases our results toward the null hypothesis, leading to underestimates of the CDI incidence associated with family exposure. When family members are covered under different policies, we are unable to detect family exposure in our nonexposed group. When enrollees live apart, familial exposures do not occur, leading to an underestimation of the association of familial exposures on the incidence of CDI.

Another limitation is that our insurance claims data do not provide details necessary to determine attributable risk (eg, complete household size, exact exposure period, date when symptoms first occurred, or other possible CDI exposures). Other factors that may be associated with household CDI risk cannot be ruled out. For example, certain genetic or household environmental factors (eg, inflammatory bowel disease, shared bathrooms) may potentially confound our estimates. Future investigations may attempt to control for other household factors.

Our study has other limitations. First, we depend on administrative claims data and do not have laboratory results. For inpatient CDI cases, we also do not know the day when symptoms or diagnosis occurred. However, our sensitivity analysis (ie, requiring subsequent hospitalization or receipt of vancomycin or metronidazole to confirm each CDI diagnosis) yielded consistent results. Second, because we are not able to perform whole genome sequencing, we are not able to confirm if CDI cases within families represent identical genetic strains. Investigations within hospital settings have suggested that some CDI cases are associated with asymptomatic carriers rather than direct transmission from another symptomatic CDI case.^34,35^

## Conclusions

Our results suggest that sharing a household with a family member with CDI is associated with the risk for acquiring CDI, even after controlling for other risk factors. Although the absolute risk of CDI following family exposure is low compared with other established risk factors, family exposure confers a high degree of relative risk. Thus, our results may have implications for highlighting the role of the environment in the spread of CDI as well as practical implications for preventing the spread of CDI in the household setting.

## Notes

Supplement. **eTable 1.** Results Using CDI Cases Validated Through Antibiotic Treatment or Subsequent CDI Hospitalization **eTable 2.** Results for Hospital Onset CDI Cases
