# Spelling Error in Author Name

**DOI:** 10.1001/jamanetworkopen.2020.16196

**Published:** 2020-07-23

In the Original Investigation titled “Association of Household Exposure to Primary *Clostridioides difficile* Infection With Secondary Infection in Family Members,”^1^ published on June 26, 2020, there was a spelling error in the author byline. The name given as “Sriram V. Pemmeraju, PhD,” should have been “Sriram V. Pemmaraju, PhD.” This article has been corrected.
