# Supplementary material for: Association of Household Exposure to Primary Clostridioides difficile Infection With Secondary Infection in Family Members
Source: JAMA Netw Open. 2020 Jun 26;3(6):e208925. doi: 10.1001/jamanetworkopen.2020.8925 (PMC7320299; doi:10.1001/jamanetworkopen.2020.8925)
Supplement: Supplement. — eTable 1. Results Using CDI Cases Validated Through Antibiotic Treatment or Subsequent CDI Hospitalization eTable 2. Results for Hospital Onset CDI Cases [file jamanetwopen-3-e208925-s001.pdf]

Supplementary Online Content

Miller AC, Segre AM, Pemmeraju SV, Sewell DK, Polgreen PM. Association of household exposure to primary *Clostridioides difficile* infection with secondary infection in family members. *JAMA Netw Open*. 2020;3(6):e208925. doi:10.1001/jamanetworkopen.2020.8925

eTable 1. Results Using CDI Cases Validated Through Antibiotic Treatment or Subsequent CDI Hospitalization

eTable 2. Results for Hospital Onset CDI Cases

This supplementary material has been provided by the authors to give readers additional information about their work.

© 2020 Miller AC et al. *JAMA Network Open*.

Supplementary Material: Validation Results

eTable 1. Results using CDI cases validated through antibiotic treatment or subsequent CDI hospitalization. In total 142,090 CDI cases occurred in inpatient settings or were validated by outpatient treatment or hospitalization. A total of 51,878 of the 134,606 community-onset CDI cases were validated by subsequent antibiotic treatment or hospitalization.

Coefficient	Any CDI (N CDI Cases = 142,090)		Community Onset CDI (N CDI Cases = 51,878)		Community Onset CDI and No Prior Hospitalization (N CDI Cases = 39,761)	
	Estimate	95% CI	Estimate	95% CI	Estimate	95% CI
**(Intercept)**	0	(0-0)	0	(0-0)	0	(0-0)
**Family Exposure**	8.351	(5.097-12.768)	10.689	(6.464-16.459)	13.261	(6.803-22.93)
**Family Exposure: dependent (age 18-26)**	0.953	(0.034-5.169)	1.405	(0.135-5.825)	1.129	(0.055-6.048)
**Prior Hospitalization**	16.741	(15.733-17.806)	9.934	(9.178-10.743)	N/A	
**Prior antibiotic use**						
None	(reference)		(reference)		(reference)	
Low-risk antibiotic	3.902	(3.607-4.215)	5.829	(5.319-6.379)	6.783	(6.012-7.636)
High-risk antibiotic	10.24	(9.59-10.929)	19.071	(17.682-20.565)	30.445	(27.682-33.475)
**Age Group**						
0-17	(reference)		(reference)		(reference)	
18-26	1.796	(1.565-2.059)	2.403	(2.034-2.839)	2.538	(2.067-3.118)
27-44	2.243	(2.006-2.512)	3.074	(2.672-3.547)	3.214	(2.701-3.842)
45-64	4.534	(4.109-5.015)	4.818	(4.237-5.5)	4.732	(4.035-5.584)
≥65	10.717	(9.657-11.916)	14.066	(12.314-16.125)	13.771	(11.644-16.372)
**Female sex**	1.209	(1.149-1.272)	1.375	(1.292-1.464)	1.404	(1.292-1.527)
**Year**						
2001	(reference)		(reference)		(reference)	
2002	1.37	(0.919-2.084)	1.103	(0.66-1.886)	1.069	(0.513-2.331)

© 2020 Miller AC et al. *JAMA Network Open*.

2003	1.414	(0.969-2.116)	1.077	(0.662-1.807)	1.033	(0.517-2.194)
2004	1.737	(1.216-2.555)	1.36	(0.868-2.22)	1.267	(0.67-2.597)
2005	1.944	(1.372-2.842)	1.617	(1.049-2.608)	1.564	(0.852-3.147)
2006	1.962	(1.39-2.861)	1.788	(1.169-2.867)	1.711	(0.944-3.414)
2007	2.256	(1.606-3.276)	1.762	(1.155-2.821)	1.712	(0.949-3.405)
2008	2.614	(1.875-3.771)	2.021	(1.341-3.204)	2.035	(1.152-3.995)
2009	2.709	(1.946-3.905)	2.543	(1.698-4.014)	2.542	(1.451-4.962)
2010	2.82	(2.025-4.066)	2.886	(1.928-4.552)	2.969	(1.698-5.786)
2011	3.292	(2.371-4.736)	3.409	(2.288-5.362)	3.576	(2.057-6.943)
2012	3.586	(2.585-5.155)	3.88	(2.607-6.095)	4.087	(2.356-7.923)
2013	3.684	(2.652-5.301)	4.005	(2.687-6.299)	4.244	(2.442-8.238)
2014	3.966	(2.856-5.706)	4.392	(2.948-6.904)	4.711	(2.714-9.138)
2015	4.261	(3.056-6.148)	5.65	(3.788-8.891)	6.239	(3.59-12.112)
2016	4.487	(3.219-6.474)	6.518	(4.375-10.247)	7.239	(4.171-14.039)
2017	4.121	(2.944-5.967)	5.983	(4-9.435)	6.574	(3.77-12.79)
**Month**						
January	(reference)		(reference)		(reference)	
February	0.709	(0.627-0.802)	0.594	(0.51-0.69)	0.557	(0.456-0.68)
March	0.585	(0.518-0.66)	0.476	(0.411-0.552)	0.443	(0.364-0.539)
April	0.575	(0.509-0.65)	0.47	(0.405-0.546)	0.443	(0.364-0.54)
May	0.569	(0.503-0.643)	0.472	(0.407-0.548)	0.444	(0.364-0.541)
June	0.592	(0.523-0.669)	0.503	(0.433-0.583)	0.471	(0.386-0.575)
July	0.591	(0.522-0.668)	0.491	(0.423-0.571)	0.479	(0.393-0.584)
August	0.606	(0.536-0.684)	0.504	(0.433-0.585)	0.477	(0.391-0.582)
September	0.613	(0.541-0.693)	0.522	(0.449-0.606)	0.508	(0.416-0.619)
October	0.605	(0.535-0.684)	0.512	(0.441-0.594)	0.49	(0.402-0.597)
November	0.578	(0.51-0.655)	0.48	(0.412-0.559)	0.468	(0.383-0.572)
December	0.572	(0.505-0.647)	0.464	(0.399-0.54)	0.433	(0.354-0.53)

© 2020 Miller AC et al. *JAMA Network Open*.

eTable 2. Results for Hospital Onset CDI cases

Coefficient	Hospital Onset CDI		Hospital Onset CDI (no prior hospitalization)	
	Estimate	95% CI	Estimate	95% CI
**(Intercept)**	0	(0-0)	0	(0-0)
**Family Exposure**	6.733	(3.299-11.995)	8.868	(3.561-17.885)
**Family Exposure: dependent (age 18-26)**	0.23	(0-6.57)	0.252	(0-9.561)
**Age Group**				
0-17	(reference)		(reference)	
18-26	1.527	(1.289-1.805)	1.667	(1.316-2.105)
27-44	1.874	(1.637-2.148)	2.195	(1.817-2.659)
45-64	4.416	(3.935-4.97)	4.772	(4.058-5.645)
≥65	9.253	(8.179-10.495)	18.123	(15.348-21.522)
**Female sex**	1.12	(1.053-1.191)	1.162	(1.067-1.266)
**Prior antibiotic use**				
None	(reference)		(reference)	
Low-risk antibiotic	3.083	(2.792-3.398)	3.757	(3.276-4.291)
High-risk antibiotic	6.973	(6.416-7.572)	13.795	(12.385-15.341)
**Prior Hospitalization**	22.49	(20.90-24.20)	N/A	
**Year**				
2001	(reference)		(reference)	
2002	1.512	(0.943-2.5)	1.481	(0.796-2.902)
2003	1.59	(1.017-2.582)	1.519	(0.844-2.911)
2004	1.931	(1.265-3.079)	1.796	(1.033-3.363)
2005	2.115	(1.397-3.353)	1.958	(1.14-3.638)
2006	2.047	(1.356-3.239)	1.884	(1.101-3.492)
2007	2.505	(1.672-3.942)	2.249	(1.329-4.136)
2008	2.912	(1.961-4.551)	2.552	(1.529-4.648)

© 2020 Miller AC et al. *JAMA Network Open*.

2009	2.782	(1.874-4.348)	2.472	(1.482-4.5)
2010	2.774	(1.867-4.338)	2.526	(1.512-4.601)
2011	3.22	(2.174-5.022)	2.908	(1.75-5.277)
2012	3.42	(2.311-5.332)	3.134	(1.89-5.683)
2013	3.502	(2.362-5.468)	3.204	(1.927-5.819)
2014	3.732	(2.518-5.824)	3.354	(2.018-6.089)
2015	3.503	(2.345-5.499)	3.211	(1.91-5.875)
2016	3.392	(2.267-5.333)	3.086	(1.831-5.656)
2017	3.136	(2.082-4.952)	2.964	(1.744-5.463)
**Month**				
January	(reference)		(reference)	
February	0.773	(0.666-0.898)	0.719	(0.587-0.88)
March	0.648	(0.559-0.751)	0.6	(0.491-0.733)
April	0.636	(0.547-0.738)	0.596	(0.486-0.73)
May	0.623	(0.537-0.723)	0.58	(0.473-0.71)
June	0.64	(0.55-0.743)	0.603	(0.491-0.74)
July	0.646	(0.557-0.75)	0.612	(0.499-0.75)
August	0.663	(0.572-0.77)	0.636	(0.519-0.778)
September	0.662	(0.569-0.769)	0.628	(0.511-0.77)
October	0.657	(0.566-0.762)	0.625	(0.51-0.765)
November	0.633	(0.544-0.737)	0.607	(0.493-0.745)
December	0.634	(0.546-0.737)	0.611	(0.498-0.748)

© 2020 Miller AC et al. *JAMA Network Open*.
